# Endoplasmic Reticulum Stress Mediates Renal Tubular Vacuolation in BK Polyomavirus-Associated Nephropathy

**DOI:** 10.3389/fendo.2022.834187

**Published:** 2022-04-08

**Authors:** Guo-Dong Zhao, Rong Gao, Xiao-Tao Hou, Hui Zhang, Xu-Tao Chen, Jin-Quan Luo, Hui-Fei Yang, Tong Chen, Xue Shen, Shi-Cong Yang, Cheng-Lin Wu, Gang Huang

**Affiliations:** ^1^ Department of Organ Transplant, The First Affiliated Hospital of Sun Yat-sen University, Guangzhou, China; ^2^ Department of Endocrinology and Metabolism, Guangdong Provincial Key Laboratory of Diabetology, The Third Affiliated Hospital of Sun Yat-sen University, Guangzhou, China; ^3^ Department of Renal Pathology, King Medical Diagnostics Center, Guangzhou, China; ^4^ Department of Pathology, Fuda Cancer Hospital·Jinan University, Guangzhou, China; ^5^ Department of Pathology, The First Affiliated Hospital of Sun Yat-sen University, Guangzhou, China

**Keywords:** BK polyomavirus, Cytoplasmic vacuolation, Endoplasmic reticulum stress, DDIT3, Drug repurposing

## Abstract

**Objective:**

This study aimed to explore the molecular mechanism of cytoplasmic vacuolation caused by BK polyomavirus (BKPyV) and thus search for potential target for drug repurposing.

**Methods:**

Morphological features of BK polyomavirus-associated nephropathy (BKPyVAN) were studied under light and electron microscopes. Microarray datasets GSE75693, GSE47199, and GSE72925 were integrated by ComBat, and differentially expressed genes (DEGs) were analyzed using limma. Furthermore, the endoplasmic reticulum (ER)-related genes obtained from GenCLiP 2.0 were intersected with DEGs. GO and KEGG enrichment pathways were performed with intersection genes by R package clusterProfiler. The single-cell RNA sequencing (scRNA-seq) from a BKPyVAN recipient was analyzed with a dataset (GSE140989) downloaded from Gene Expression Omnibus (GEO) as control for gene set variation analysis (GSVA). Immunohistochemistry and electron microscopy of kidney sections from drug-induced ERS mouse models were performed to explore the association of ERS and renal tubular vacuolation. Protein–protein interaction (PPI) network of the intersection genes was constructed to identify hub target. AutoDock was used to screen Food and Drug Administration (FDA)-approved drugs that potentially targeted hub gene.

**Results:**

Light and electron microscopes exhibited obvious intranuclear inclusions, vacuoles, and virus particles in BKPyV-infected renal tubular cells. Transcriptome analysis revealed 629 DEGs between samples of BKPyVAN and stable transplanted kidneys, of which 16 were ER-associated genes. GO analysis with the intersection genes illustrated that ERS-related pathways were significantly involved, and KEGG analysis showed a prominent enrichment of MAPK, Toll-like receptor, and chemokine signaling pathways. GSVA analysis of the proximal tubule revealed similar pathways enrichment. An electron microscope image of the kidney from ERS mouse models showed an obvious renal tubular vacuolation with prominent activation of ERS markers verified by immunohistochemistry. Furthermore, DDIT3 was identified as the hub gene based on PPI analysis, and ZINCOOOOO1531009 (Risedronate) was indicated to be a potential drug for DDIT3.

**Conclusion:**

ERS was involved in renal tubular cytoplasmic vacuolation in BKPyVAN recipients. Risedronate was screened as a potential drug for BKPyVAN by targeting DDIT3.

## Introduction

BK polyomavirus (BKPyV) is a kind of small (~50.0 nm in diameter) circular, non-enveloped, double-stranded DNA polyomavirus with icosahedral symmetry (1). BKPyV, which establishes a lifelong persistent infection, is widespread, and more than 80% of adults worldwide have been infected, which occurs in early childhood (2). The initial BKPyV infection generally occurs in tonsils (3) and gradually spreads to other tissues and organs, especially the urinary system (4). In general, clinical signs and symptoms of BKPyV infection are inconspicuous. In kidney transplant recipients, BKPyV may reactivate and cause BK polyomavirus-associated nephropathy (BKPyVAN) with an incidence of 1%–10% (5). BKPyVAN is a main risk factor for renal allograft dysfunction after transplantation. Up to 50% of renal allograft loss was caused by polyomavirus associated nephropathy (6). At present, specific anti-BKPyV drug is not available. Reducing the dose of immunosuppressive drugs is still the primary way to control BKPyV replication. In this way, it is more susceptible to suffer from transplant rejection. Therefore, it is urgent to explore pathogenesis of BKPyVAN.

The endoplasmic reticulum (ER) is known to be a vital organelle involve in protein folding, translocation, and post-translational modification in eukaryotic cells. Because of the large and complex membrane structure with massive functional proteins attachment to the surface, it is critical to multiple signaling pathways regulation. In addition, only the mature and correctly folded protein in the ER can be transported to the Golgi apparatus and become a secreted protein or enter the endocytic pathway (7). When the folding of peptide chains in the ER is blocked, massive misfolded proteins accumulate, which puts the cells under state of stress, called ER stress (ERS) (8). Recently, great breakthroughs have been made in the study of the mechanism of viral infection and ERS. In the early stage, mild ERS can mitigate the damage, but excessive or long-term stress can induce apoptosis, which facilitates virus replication and release of virus particles (9). BKPyV, in common with most virus, relies on the ER to produce productive infection by synthesizing protein (10). Previous studies on BKPyV and ER have revealed the involvement of intracellular trafficking pathways (11). There are still many gaps, however, in our knowledge of ERS-specific pathological changes of BKPyVAN and whether ERS plays a critical role in the production and progression of BKPyVAN.

In this study, we comprehensively explored the essential role of ERS in the BKPyVAN recipients, combined with omics analysis and experimental verification, and further identified DDIT3 as a hub gene involving in this process. Our report put forward a new viewpoint into the pathology of BKPyVAN and provided a potential therapeutic target for the treatment of BKPyVAN.

## Subjects and Methods

### ERS of Mouse Models

Male C57BL/6J mice aged 8–10 weeks were provided by Guangdong Laboratory Animals Monitoring Institute. Three mice were intraperitoneally injected with 3 mg/kg tunicamycin (Sigma, Shanghai, China) to induce ERS (12), and another three mice were intraperitoneally injected with equal doses of dimethyl sulfoxide (Sigma, Shanghai, China) as negative controls. After 6 h, mice were sacrificed, and kidneys were removed immediately. Appropriate volume of the kidney was immediately cut off and put into fixative fluids for further exploration.

### Human Samples

Kidney samples from five BKPyVAN recipients, one stable transplanted recipient and one resolved BKPyVAN (by reducing immunosuppressants, viremia changed from positive to negative), were obtained in this study. They were used to conduct pathological research. Besides, one of the BKPyVAN recipients (BKPyVAN recipient 5) was processed to generate the single-cell RNA sequencing (scRNA-seq) profile. Detailed information about all samples was provided in **Supplementary Table S1**. Ethical approvals were obtained from the ethics committee of the First Affiliated Hospital of Sun Yat-sen University.

### Pathological Methods

Specimens were fixed in 10% neutral buffered formalin, embedded in paraffin, sectioned at 4-μm thickness, and further processed according to standard hematoxylin and eosin (H&E) staining protocol. Pathological diagnosis was confirmed by immunostaining with antibody against SV40 large T-antigen (#DP02, Oncogene Research Products, Cambridge, MA, USA) as previously described (13).

Electron microscopy was used to detect viral particles and ultrastructure, especially in tubular epithelial cells. Kidney tissues were fixed in 2.5% glutaraldehyde (A17876, Ala Aesar, Heysham, LRE; UK) and 1% osmic acid (#18456, Ted Pella, Altadena, CA,USA) in turn. Next, dehydration and resin embedding were performed. Semi-thin sections (1,000 nm thick) were stained with toluidine blue, and the renal tubules (to be located if there are intranuclear inclusion body epithelial cells) and glomerulus were observed and located under a light microscope. Ultrathin sections (50–70 nm thick) were performed. After double staining of 2% uranyl acetate (#22400, EMS, Hatfield, PA, USA) and 3% lead citrate (#19314, Ted Pella, USA), transmission electron microscopy (JEM-1400 PLUS, Japan Electron Optics Laboratory Co., Ltd, Akishimashi, TKY, JPN) was used to observe.

### Data of Bulk RNA Sequence

The GSE75693 (14), GSE47199 (15), and GSE72925 (16) datasets, with a total of 137 samples including 28 BKPyVAN recipients and 109 stable transplanted kidneys, were downloaded from GEO. R package Combat was used to eliminate batch effects. The differentially expressed genes (DEGs) of BKPyVAN and stable transplanted kidneys were analyzed by R package limma. p-Value < 0.05 and | fold change (FC)| > 1.5 were considered statistically significant. The ER-related gene sets were obtained through GenCLiP 2.0 (http://ci.smu.edu.cn/GenCLiP2/analysis.php) and Gene Ontology (GO, http://geneontology.org). Finally, the Venn diagram was drawn according to the intersection of DEGs and ER-related genes.

### KEGG and GO Enrichment Analyses

GO and Kyoto Encyclopedia of Genes and Genomes (KEGG) enrichment analyses of intersection genes were performed through the ClusterProfiler (17) package in R software, and p-value < 0.05 was considered to be statistically significant.

### scRNA-seq and Functional Enrichment Analysis

scRNA-seq data of healthy transplanted kidneys were obtained from the GSE140989 (18), while data of BKPyVAN were generated from a BKPyVAN recipient in the First Affiliated Hospital of Sun Yat-sen University. Detailed information on tissue processing, 10X Genomic sample processing, and gene-set variation analysis (GSVA) was given in the **Supplementary Material**.

### Immunohistochemistry of Mouse Models

Immunohistochemistry (IHC) of anti-glucose-regulated protein 78 (GRP78) and anti-CCAAT/enhancer-binding protein (C/EBP) homologous protein [CHOP, also known as DNA damage inducible transcript 3 (DDIT3)/GADD153], which were widely regarded as ERS markers (19, 20), was performed on kidney tissues of mouse models. A series of steps were performed, including paraffin sectioning, dewaxing, and antigen retrieval (3% H_2_O_2_, 37°C, 10 min, and sodium citrate, 100°C, 30 min). After blocking for 20 min at room temperature, sections were incubated overnight at 4°C with either anti-GRP78 antibody (1:2,000, ab21685, Abcam, Cambridgeshire, United Kingdom) or anti-CHOP antibody (1:100, #2895, Cell Signaling Technology, Danvers, MA, United States), followed by the incubation with secondary antibody for 60 min at room temperature. Finally, the sections were developed with 3,31-diaminobenzidine (DAB).

### Protein–Protein Interaction Network Construction and Hub Gene Screening

The intersection genes were imported into the STRING 11.5 database (21) to obtain the protein–protein interaction (PPI) network. The hub gene was analyzed using cytoHubba through five algorithms: Degree, EcCentricity, Stree, Betweenness, and MNC.

### Virtual Drug Screening Based on the Structure of Core Gene

The 3D structure of hub gene was downloaded from Protein Data Bank (PDB, https://www.rcsb.org/), and the active site is predicted by DoGSiteScorer (22). Thereafter, a small molecules database of 2,106 FDA-approved drugs downloaded from the ZINC15 database (23) was established. Finally, AutoDock 4.0 (24) was used for virtual screening and molecular docking.

## Results

### Morphological Changes

**Figure 1:**
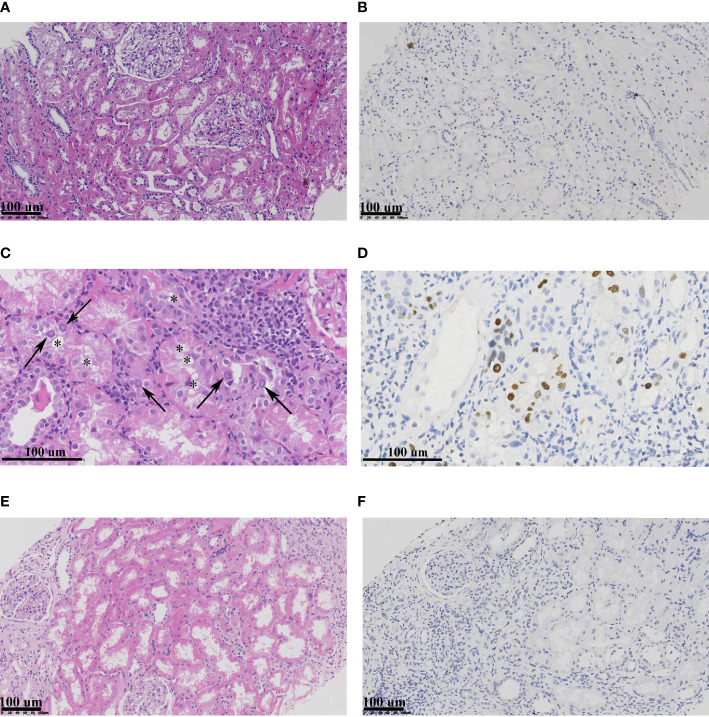
The light micrographs of samples. Compared with stable transplanted recipient **(A, B)** and resolved BKPyVAN **(E, F)**, the epithelial cells were swollen with large vacuole (*) within the cytoplasm, and typical intranuclear inclusion (arrows) can be observed in the BKPyVAN recipient 1 **(C, D)**. In addition, anti-SV40 T-Ag positivity in tubular epithelial cell nuclei. Immunohistochemistry staining (IHC) of panels **(B, D, F)** corresponding to HE staining **(A, C, E)** (HE), respectively.

To explore the morphological changes of kidney infected by BKPyV, light and electron microscopy were performed. Obvious cytoplasmic vacuoles (**Figure 1C** and **Supplementary Figures S1A, C, E, G**) could be observed in the renal tubular epithelial cells from BKPyVAN recipients, paralleling to positive SV40 T-Ag in the nucleus as shown in IHC (**Figure 1D** and **Supplementary Figures S1B, D, F, H** ), when compared to that of stable transplanted recipients (**Figures 1A, B** **)** and resolved BKPyVAN (**Figures 1E, F** **)**.

**Figure 2:**
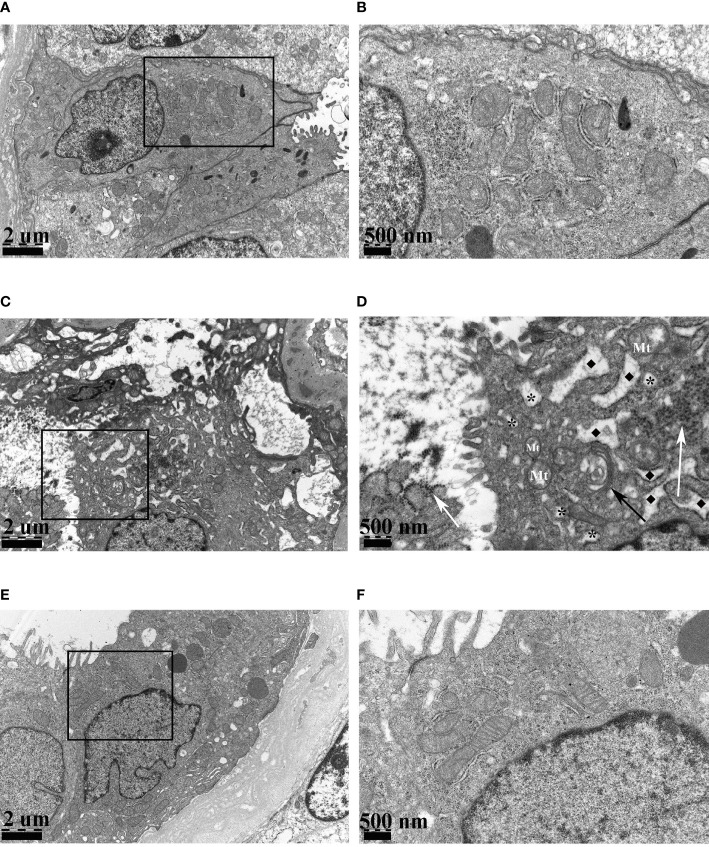
The electron micrographs of stable transplanted recipient **(A, B)**, BKPyVAN recipient 2 **(C, D)**, and resolved BKPyVAN **(E, F)**. The figures of the high-power field on the right **(B, D, F)** corresponded to the figures in the box of the low-power field on the left **(A, C, E)**. The mild swelling of mitochondrion (Mt) and Golgi apparatus (black arrow), numerous virus particles (white arrows), cytoplasmic vacuolar degeneration (*), and expansion of rough endoplasmic reticulum (◆) can be observed in BKPyVAN. **(A, C, E)** × 12,000, **(B, D, F)** × 30,000.

Under the electron microscope, compared to stable transplanted recipients (**Figures 2A, B** **)**, virus particles with a diameter of about 35 nm were observed in BKPyVAN recipients. They were distributed in clusters and in the cytoplasm of tubular epithelial cell and renal tubular lumen. In addition to the characteristic ultrastructural features of BKPyV, mild swelling and focal vacuolar degeneration of mitochondrion and mild swelling of Golgi apparatus were observed. More obviously, we observed that rough ER proliferated, expanded, and occurred in partial degranulation (**Figures 2C, D** and **Supplementary Figures S2A–D**). The BKPyVAN recipients suffered obvious tubulitis. In addition, necrotic and disintegrated cell fragmentation and apoptotic cells can be observed in the lumen (**Figures S2C, D**). For resolved BKPyVAN, however, we did not observe the expansion of ER and vacuoles (**Figures 2E, F** **)**.

### Identification of DEGs and Gene Pathway Analysis

**Figure 3:**
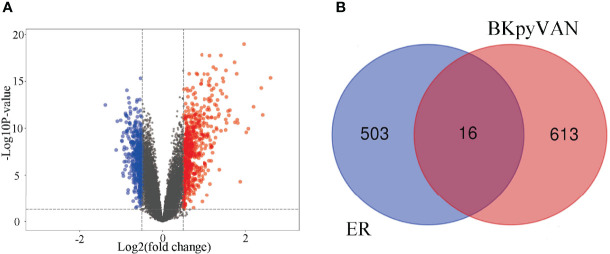
Prediction of BKPyV-related genes and endoplasmic reticulum-related genes. **(A)** Volcano plot shows that differentially expressed genes (DEGs) between BKPyVAN and stable transplanted kidney [BKPyVAN=28, stable transplanted kidney=109, p-value < 0.01 and | fold change (FC)| > 1.5]. The blue part indicates the downregulated genes. The red part represents the upregulated genes. The black part shows the stable genes. **(B)** Venn diagram identifies genes related to BKPyVAN and endoplasmic reticulum.

**Figure 4:**
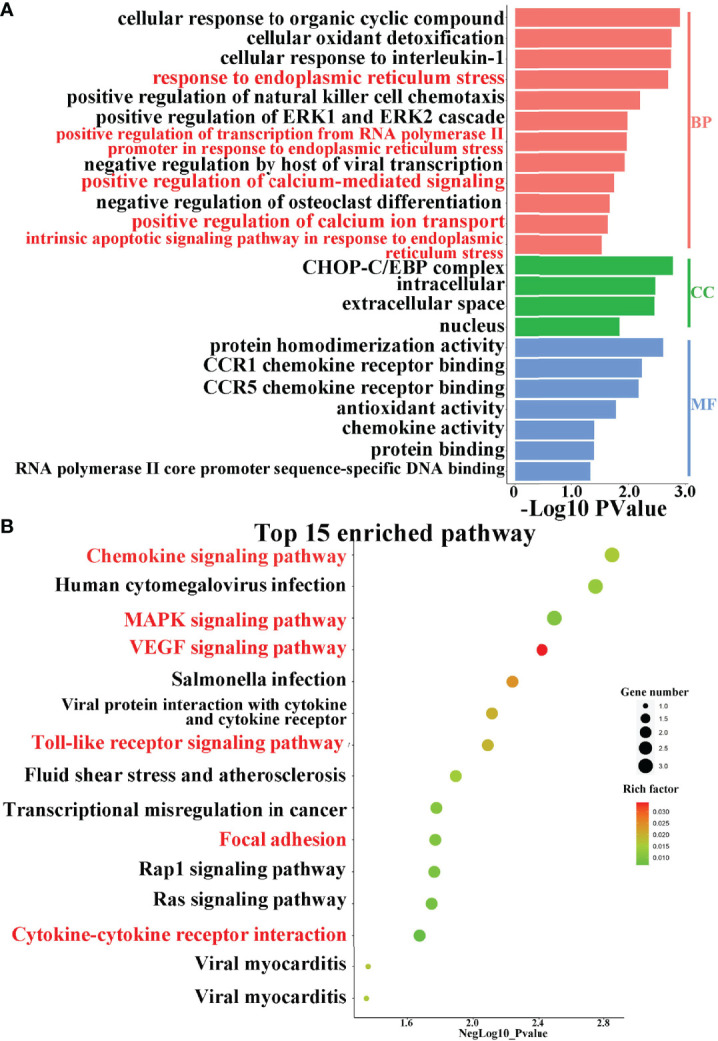
Pathway enrichment analysis. **(A)** GO enrichment analysis of intersection genes. GO analysis divided gens into three functional groups: molecular function (MF), biological processes (BP), and cell composition (CC). **(B)** Dot plot of KEGG enrichment analysis. The abscissa indicates the -Log10_Pvalue, the ordinate indicates the names of the pathways. The size of the dot indicates the number of enriched genes, and the different colors indicate the rich factor.

To explore the possible mechanism of ER involvement in BKPyVAN recipients, we analyzed an RNA-seq dataset including 28 recipients with BKPyVAN and 109 individuals with stable transplanted kidney. A total of 629 DEGs (**Figure 3A**) between BKPyVAN recipients and stable transplanted kidney were acquired, of which 16 were ER-related genes (**Figure 3B**). As summarized in **Figure 4A**, GO analysis was performed to reveal a prominent involvement of ERS pathways, including “response to endoplasmic reticulum stress”, “positive regulation of transcription from RNA polymerase II promoter in response to endoplasmic reticulum stress”, “positive regulation of calcium-mediated signaling”, “positive regulation of calcium ion transport”, and “intrinsic apoptotic signaling pathway in response to endoplasmic reticulum stress”. In **Figure 4B**, KEGG analysis showed top 15 pathways of significant enrichment. Among them, six pathways were closely related with ERS and ranked relatively high, including “chemokine signaling pathway”, “MAPK signaling pathway”, “VEGF signaling pathway”, “Toll-like receptor signaling pathway”, “cytokine–cytokine receptor interaction”, and “focal adhesion”. Other pathways were mainly related to infection, tumor, and atherosclerosis, including “human cytomegalovirus infection,” “viral protein interaction with cytokine and cytokine receptor”, “Rap1 signaling pathway”, “Ras signaling pathway”, and “fluid shear stress and atherosclerosis”.

### GSVA of scRNA-seq

**Figure 5:**
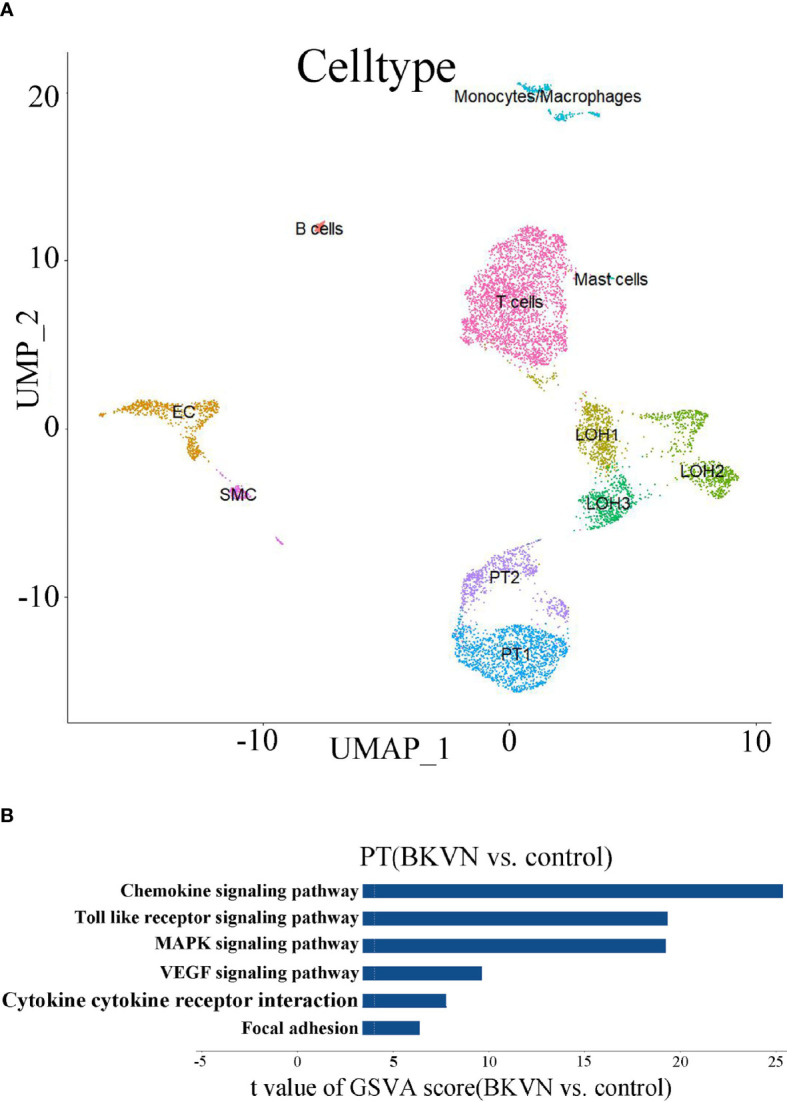
scRNA analysis of PT in the kidney. **(A)** UMAP plot of cell clusters identified on the basis of the expression of highly variable genes (at a resolution of 0.25). **(B)** Bar plot of the differences in six pathways between BKPyVAN and healthy transplanted kidney (n = 3,275 and 5,401 cells from six samples, respectively). Resulting from the GSVA score comparison tests comparing BKPyVAN with healthy controls.

We observed the obvious vacuolar degeneration of renal tubules in BKPyVAN recipients. In order to further explore their pathways that may be involved in the renal tubular epithelial cells during BKPyV infection, we carried out scRNA-seq. Initially, the scRNA-seq data of BKPyVAN recipients and healthy transplanted kidney were integrated and corrected by R package Harmony in order to eliminate the batch effect. We defined 11 clusters of specific cell types at a resolution of 0.25 (**Figure 5A**), as follows: T cells (TRBC2), proximal tubule (PT) cells (ALDOB), loop of Henle (LOH) (UMOD, SLC12A1), epithelial cells (EC) (ENG, EMCN), monocytes/macrophages (PLAUR, CD74), smooth muscle cells (SMCs) (TAGLN), B cells (CD79A, CD79B, CD74), and mast cells (TPSAB1, TPSB2, CPA3, MS4A2). Additionally, we used a heatmap to visualize the marker genes related to all clusters to confirm the representativeness of our cell-type classification (**Supplementary Figure S3**).

In order to further exert the advantages of scRNA-seq, we chose PT cells for further GSVA. Similar to the results of KEGG, we found that “MAPK signaling pathway”, “Toll-like receptor signaling pathway”, “chemokine signaling pathway”, “cytokine–cytokine receptor interaction”, “VEGF signaling pathway”, and “focal adhesion” were significantly enriched, and all of them were upregulated in BKPyVAN recipients (**Figure 5B**). This further showed that BKPyV caused renal tubular ERS and participated in renal tubular disease. Complete result of GSVA is shown in **Supplementary Table 2**.

### Immunohistochemistry and Microscopic Observation of Mouse Models

**Figure 6:**
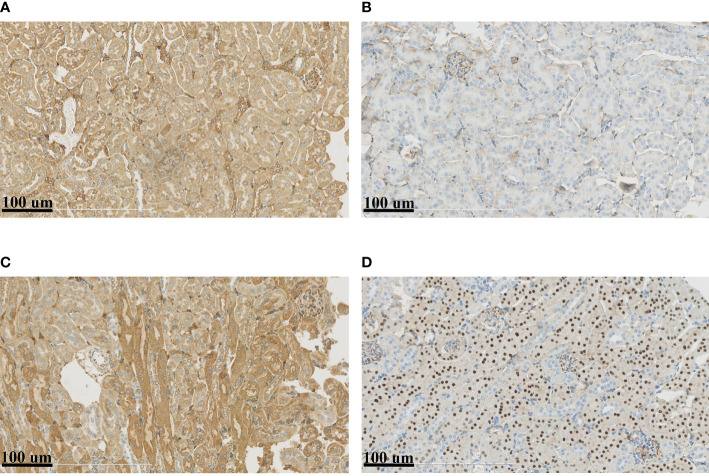
Immunohistochemistry of mouse kidney models. Compared to control **(A, B)**, the immunohistochemistry of ERS mouse model **(C, D)** showed that the expression of GRP78 **(A, C)** and CHOP **(B, D)** were increased.

**Figure 7:**
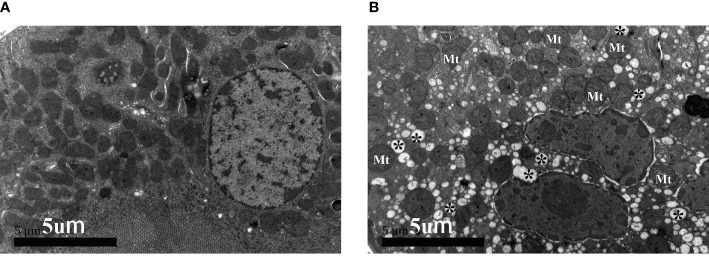
The electron micrographs of control **(A)** and mouse kidney model of ERS **(B)**. Swelling of mitochondrion (Mt) and cytoplasmic vacuolar degeneration (*) can be observed in ERS mouse model.

The above results suggested that the renal tubular epithelial cells of BKPyVAN recipients suffered ERS. In order to better illustrate the role of the ERS in renal tubular vacuolation of BKPyVAN recipients, we used mouse models of ERS. We observed that the expression of GRP78 was increased, especially in the area of corticomedullary junction, and CHOP presented obvious nuclear positioning in mice injected with tunicamycin (**Figures 6C, D** **)**, compared with control (**Figures 6A, B** **)**, indicating that we used successful mouse models of ERS. In addition, compared with control (**Figure 7A**), mild swelling of the mitochondrion and a large amount of vacuoles were observed in the cytoplasm under electron microscopy (**Figure 7B**), very similar to what we described above for BKPyVAN. It suggested that ERS can mediate cytoplasmic vacuolation, and a similar mechanism may be present in BKPyVAN recipients.

### Construction of Protein–Protein Interaction Network and Screening of Candidate Gene

**Figure 8:**
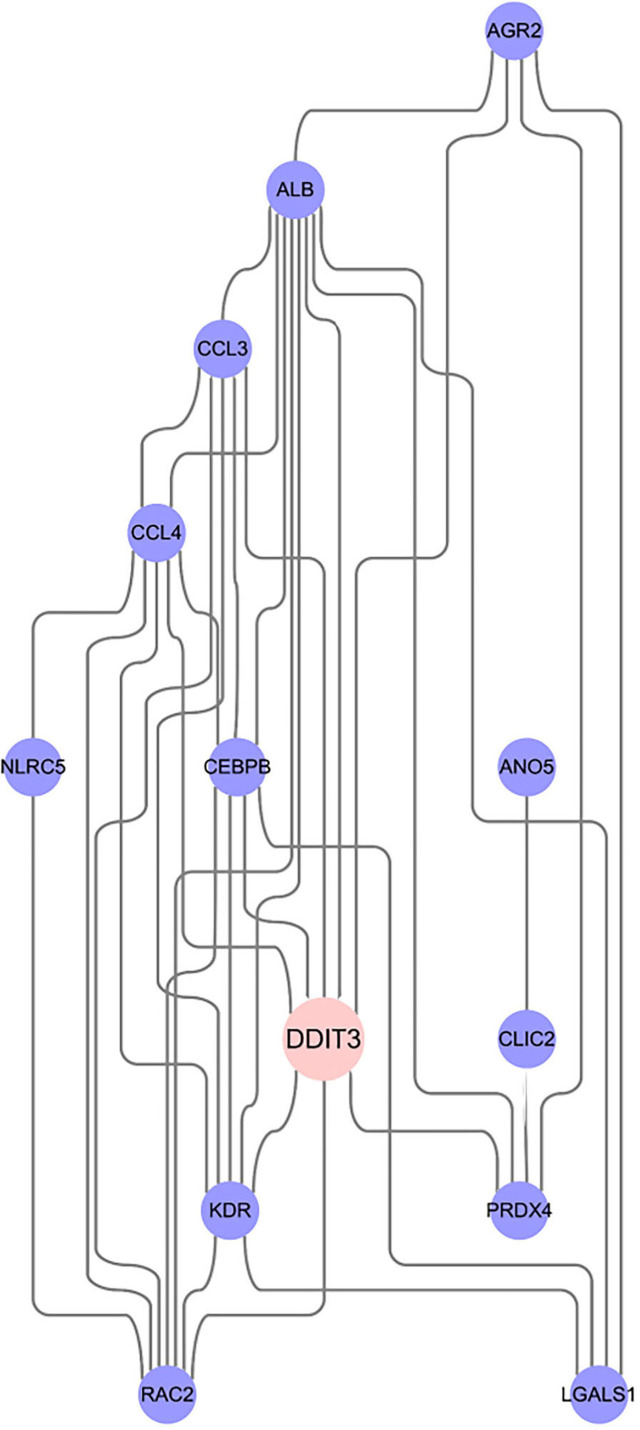
Determine candidate gene according to the PPI network. PPI network diagram of intersection genes.

As shown in **Figure 8**, the PPI network was composed of 13 nodes and 34 edges. Each node represented a gene, and each edge represented two genes contributing an identical function. In the network, DDIT3 had the most edges. Through the retrieval in Genecards (https://www.genecards.org/), we found that eight genes related to DDIT3 respectively encoded chemokine (CXCL3 and CXCL4), transcription factor (CEBPB), disulfide isomerase family of ER proteins (AGR2), antioxidant enzyme (PRDX4), Ras superfamily of small guanosine triphosphate GTP-metabolizing proteins (RAC2), receptor of the VEGF (KDR), and albumin (ALB). DDIT3 got the highest score by algorithm (**Table 1**). Thus, we identified it as hub gene, which might be of importance in the pathogenesis of BKPyV-induced ERS.

**Table 1:** Intersection genes evaluated by BottleNeck and EcCentricity in the PPI.

Node name	BottleNeck (n)	EcCentricity (score)
DDIT3	10	0.33
PRDX4	3	0.33
CEBPB	2	0.25
RAC2	2	0.25
CLIC2	2	0.25
ALB	1	0.33
KDR	1	0.25
CCL4	1	0.25
CCL3	1	0.25
AGR2	1	0.33
LGALS1	1	0.25
NLRC5	1	0.20
ANO5	1	0.20

### Drug Screening and Molecular Docking

**Figure 9:**
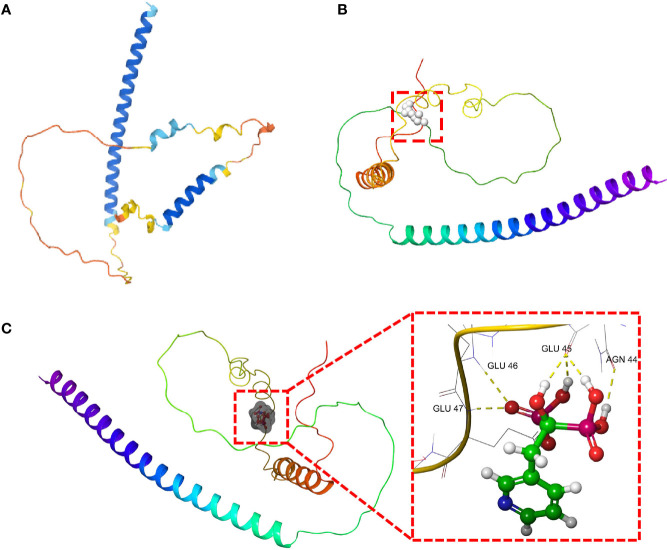
The molecular docking of Risedronate with DDIT3. **(A)** The 3D structure of DDIT3 protein from AlphaFold. **(B)** The active site of the DDIT3 protein predicted by DoGSiteScore. **(C)** The docking result of Risedronate and DDIT3.

In order to further guide the clinical treatment, drug screening was performed. We used the virtual screening technology of AutoDock to determine potentially effective drug for DDIT3. The 3D protein structure of DDIT3 was downloaded from PDB (**Figures 9A, B** **)**. According to the docking score, ZINCOOOOO1531009 (Risedronate) was the most related drug. The binding between Risedronate and DDIT3 was shown in **Figure 9C**. The key amino acid residues (AGN-44, GLU-45, GLU-46, and GLU-47) interacted with Risedronate to form a hydrogen bond interaction. Risedronate may affect the function of DDIT3 by activating or inhibiting its biological activity center, which may have potential value.

## Discussion

Our study has confirmed that the tubule epithelial cells underwent vacuolar degeneration after BKPyV infection and identified important signaling pathways and hub gene through pathology and bioinformation analysis. DDIT3 was a hub gene during the BKPyV-inducing cytoplasmic vacuolation *via* ERS, and Risedronate may be a potential antiviral drug.

Vacuolation has been demonstrated for many virus families, such as hepatitis A virus (25), dengue virus (26), and human immunodeficiency virus (27). Xuan Guo et al. reported that human fetal liver stem cells infected with blood-borne HCV experienced ER swelling, mitochondrial swelling, and cell vacuolation, and the pathogenesis may be in relation to ERS and mitochondrial-related/caspase-dependent apoptosis in cells at the early stage of infection (28). The study of Blandine Monel et al. illustrated that Zika virus (ZIKV) can cause cytoplasmic vacuolation through ERS and unprogrammed cell death and further verified that vacuoles originate from the ER (9). For the polyomavirus family, the pathological features of vacuolation of SV40 (29) and JC polyomavirus (30) have been reported, but the corresponding reports of BKPyV are still insufficient, as far as we known. Our research just confirmed that BKPyV can also cause vacuolation. In our study, we revealed that BKPyV can cause ERS and cytoplasmic vacuolar degeneration mediated by ERS. Consequently, we conjectured that the specific mechanism of vacuolar degeneration, in the course of BKPyV infection, may revolve around ERS triggered by the large number of viral proteins synthesized.

We confirmed that BKPyVAN recipients suffered ERS, and ERS was involved in the formation of the vacuolation of renal tubes. KEGG analysis of bulk RNA-seq showed that six signaling pathways had the closest relationship with ERS. In addition, scRNA-seq of PT also demonstrated that they were upregulated, so we thought that they have a key connection with vacuolation after BKPyV infection. First, mitogen-activated protein kinase (MAPK), an important transmitter of signals, is mainly involved in regulating cell growth, differentiation, stress adaptation, inflammation, and other important physiological and pathological processes. Joo Hyun Lim et al. (31) demonstrated that mitochondrial dysfunction and concomitant activation of p38 MAPK increased cytoplasmic-free Ca^2+^ levels and further induced ERS in human liver sk-HepI cells. Motamedi et al. (32) have confirmed that SV40 polyomavirus, as a member of the polyomavirus family, can activate the Ras-MAPK signaling pathway to cause specific vacuolation, cell death, and virus release.

Second, unfolded protein response affects many pathways through regulating levels of cytokines, including the producing cytokines, stimulating pattern recognition receptors, and modulating inflammatory signaling pathways and cytokine transcription factors (33). Yin et al. found that reduction in CXCR3 downregulated ERS signaling pathway and level of CHOP in chondrocyte apoptosis mediated by nitric oxide (34). Weiwei Zou et al. showed that PERK-phosphorylated eIF2a pathway could negatively regulate the expression of CXCL5 (35). In addition, other cytokines also contribute to induction of ERS in multiple cell types, such as interleukin (IL)-10, nuclear factor kappa B (NF-κB), tumor necrosis factor alpha (TNF-α), and interferon gamma (IFN-γ) (36). Third, Toll-like receptors (TLRs), as a family of pattern recognition receptors, are significant for innate immunity (37). Fabio Martinon et al. reported that TLR4 and TLR2 specifically activated the ERS sensor kinase IRE1 and supported that ERS may regulate the intensity and duration of innate immune responses, which then promoted inflammation (38). TLR4 activation causes NF-κB to enter the nucleus and subsequent translation of proinflammatory cytokines (39), which can lead to ERS (40). It is obvious that the above three pathways are related to ERS and inflammation. Therefore, cytokines and TLRs may also play an important role in the interaction between BKPyVAN and the ER.

On the whole, these results indicated that the enriched signaling pathways were related to ERS. Therefore, intervention for targeting ERS-related pathways may be an important method for improving vacuolation and treating renal injury. The expression of CHOP in mouse models significantly increased, which showed that DDIT3 was clearly associated with ERS. CHOP is a downstream target in the pathways of UPR activation (41) and one of the components of the ERS-mediated apoptotic pathway (42). DDIT3 is transcribed at a very low level under normal circumstances but rises sharply and activates apoptosis in ERS (43, 44). DDIT3 activation has been taken into account as a key trigger of ERS-related apoptosis. Studies on CHOP gene deficient in both cellular and animal models have elucidated the proapoptotic effect of CHOP during cellular stress (45, 46). It can activate the intrinsic pathway of classic apoptosis, containing gene expression of the BCL2-family proteins (47). Cell death induced by ERS can also be mediated through exogenous pathways, that is, CHOP has been shown to control the transcription of the TNF family member cell-surface DR5 to further trigger caspase 8-induced apoptosis (48). In addition, DDIT3 can mediate apoptosis through other genes, such as GADD34, ERO1α, and TRB-3 (49–53).

In brief, DDIT3 mainly mediates cell apoptosis through directly or indirectly striking the balance of pro- and anti-apoptotic genes expression (51). In some anti-tumor cell experiments, certain compounds can upregulate ERS markers, including DDIT3, and induce cytoplasmic vacuolation and the cell death (54–57). Our results indicated that the BKPyV may have a similar mechanism. Although there are few relevant literature reports, it suggests that the regulation of DDIT3 may be a good strategy for controlling the replication and spread of BKPyV. The molecular docking results showed that the screened drug exhibited good affinity with the key target DDIT3, and the binding sites had stable hydrogen bonds, stable conformation, and strong binding ability. This may provide a new direction for further development and utilization of drugs.

In summary, this study combining pathology and bioinformatics observed that BKPyV infection can lead to the cytoplasmic vacuolation involving ERS. We identified the key signaling pathways and verified *in vivo* in mouse models. Finally, we identified the hub gene DDIT3 and preliminarily screened drug based on PPI. We believe that it can provide novel ideas and references for the follow-up in-depth studies of the pathological mechanism of BKPyV and potential drug.

## Data Availability Statement

The original contributions presented in the study are included in the article/**Supplementary Material**. Further inquiries can be directed to the corresponding authors.

## Ethics Statement

The studies involving human participants were reviewed and approved by Ethics Committee of The First Affiliated Hospital of Sun Yat-sen University. The patients/participants provided their written informed consent to participate in this study. The animal study was reviewed and approved by Ethics Committee of Sun Yat-sen University.

## Author Contributions

GH and C-LW designed the study and reviewed and edited the whole manuscript. G-DZ, X-TH, and RG interpreted data and wrote the manuscript. HZ and X-TC reviewed the whole manuscript. J-QL, TC, and XS collected, analyzed, and interpreted data. X-TC and RG performed laboratory testing. G-DZ and HZ conducted bioinformatics analysis. X-TH, H-FY, and S-CY, as pathologists, evaluated the slides. All authors contributed to the article and approved the submitted version.

## Funding

This work was supported by grants from the National Natural Science Foundation of China (81770749) and The National Key Research and Development Program of China (2019YFA0111500).

## Conflict of Interest

The authors declare that the research was conducted in the absence of any commercial or financial relationships that could be construed as a potential conflict of interest.

The handling editor GS declared a shared affiliation with the authors GZ, HZ, XC, JL, TC, XS, SY, CW, GH at the time of review.

## Publisher’s Note

All claims expressed in this article are solely those of the authors and do not necessarily represent those of their affiliated organizations, or those of the publisher, the editors and the reviewers. Any product that may be evaluated in this article, or claim that may be made by its manufacturer, is not guaranteed or endorsed by the publisher.

## Abbreviations

BKPyV, BK polyomavirus; BKPyVAN, BK polyomavirus-associated nephropathy; ER, endoplasmic reticulum; ERS, ER stress; MPyV, mouse polyomavirus; scRNA-seq, single-cell RNA sequencing; DEGs, differentially expressed genes; GO, Gene Ontology; KEGG, Kyoto Encyclopedia of Genes and Genomes; GSVA, gene set variation analysis; GRP78, glucose-regulated protein 78; CHOP, CCAAT/enhancer-binding protein (C/EBP) homologous protein; DDIT3, DNA damage-inducible transcript 3; PPI, protein–protein interaction; PT, proximal tubule; MAPK, mitogen-activated protein kinase; TLR, Toll-like receptors.
