# Supplementary material for: Endoplasmic Reticulum Stress Mediates Renal Tubular Vacuolation in BK Polyomavirus-Associated Nephropathy
Source: Front Endocrinol (Lausanne). 2022 Apr 8;13:834187. doi: 10.3389/fendo.2022.834187 (PMC9027570; doi:10.3389/fendo.2022.834187)
Supplement: Supplementary file 1 [file Presentation_1.pdf]

Supplementary Material

BKPyVAN tissue acquisition and processing

One fresh BKPyVAN sample was obtained through renal graft biopsy in the First Affiliated Hospital of Sun Yat-sen University. Ice was used to minimize the transcriptional degradation during the transportation.

Single cell preparation

Then, Kidney tissue (0.2-0.9g) was transferred into gentleMACS C-tubes (Miltenyi Biotec) containing enzymatic Base Solution (100 μ GML-1 Liberase TH Research Grade and 50 μ GML-1 DNAse I, HBSS 10 mM HEPES,30 mM taurine). Tissue was dissociated using scissors (FST) and gentleMACS Octo Dissociator (Miltenyi Biotec) with heaters. The digested cells were washed with base solution containing 20 percent fetal bovine serum (FBS, Gibco)and filtered through 70 μm Nylon strainer (BD Falcon). Kidney single-cell suspension were centrifuged at 330×g for 10 min at 4 °C and resuspended in base solution containing 0.2% FBS . Cells were counted three times by TrypanBlue exclusion and , finally, resuspended at a concentration of ≥ 2×106/ml.

scRNA-seq

Single cells were processed using a 10× Chromium system (10 × Genomics). Libraries, prepared by LC Sciences following the recommended protocol for the Chromium Single Cell 30 Reagent Kit (v2 Chemistry), were run on the HiSeq4000 for Illumina sequencing. 10× Cell Ranger package (v1.2.0; 10 × Genomics) was used for postprocessing and quality control. Reads were aligned to the mm10 reference assembly (v1.2.0; 10 × Genomics).

Single-Cell RNA Sequencing Data Analysis

R package Seurat (version 4.0.4)(1) was used to analyze the gene expression matrices generated by the 10× Cell Ranger aggregate option, following the instruction (https://astijalab.org/Seurat/) with default parameters. scRNA-seq of five healthy transplanted kidney (GEO) and one BKPyVAN were integrated. Cells with 200 - 2,500 unique expressed genes and the mitochondrial gene percentage < 30% were selected to further analyze. After QC, there were 8676 high-quality kidney cells. Since these data came from six different samples, R package Harmony (version 0.1.0)(2) was used to mitigate the batch effect. The identified clusters were then visualized using uniform manifold approximation and projection (UMAP). The FindAllMarkers function was used to identify differentially expressed genes (DEGs) of each cluster. scHCL (version 0.1.1)(3) was used to identify the cell types of each cluster. To assign pathway activity estimates to individual cells, the R package GSVA (version 1.40.1)(4) with the standard settings was implemented, and a gene set containing kyoto encyclopedia of genes and genomes (KEGG) pathways was downloaded from the MsigDB database(5). The pathway activity for each cell was scored with gene set variation analysis (GSVA) methods as described previously(6) Differential pathway activities between two groups were calculated using limma package (version 3.48.3)(7) and the results were visualized using bar plot.

Reference

1. Hao Y, Hao S, Andersen-Nissen E, Mauck WM, 3rd, Zheng S, Butler A, et al. Integrated analysis of multimodal single-cell data. Cell. 2021;184(13):3573-3587.doi:10.1016/j.cell.2021.04.048
2. Korsunsky I, Millard N, Fan J, Slowikowski K, Zhang F, Wei K, et al. Fast, sensitive and accurate integration of single-cell data with Harmony. Nat Methods. 2019;16(12):1289-1296.doi:10.1038/s41592-019-0619-0
3. Han X, Zhou Z, Fei L, Sun H, Wang R, Chen Y, et al. Construction of a human cell landscape at single-cell level. Nature. 2020;581(7808):303-309.doi:10.1038/s41586-020-2157-4
4. Hanzelmann S, Castelo R, Guinney J. GSVA: gene set variation analysis for microarray and RNA-seq data. BMC Bioinformatics. 2013;14:7.doi:10.1186/1471-2105-14-7
5. Liberzon A, Subramanian A, Pinchback R, Thorvaldsdottir H, Tamayo P, Mesirov JP. Molecular signatures database (MSigDB) 3.0. Bioinformatics. 2011;27(12):1739-40.doi:10.1093/bioinformatics/btr260
6. Lambrechts D, Wauters E, Boeckx B, Aibar S, Nittner D, Burton O, et al. Phenotype molding of stromal cells in the lung tumor microenvironment. Nat Med. 2018;24(8):1277-1289.doi:10.1038/s41591-018-0096-5
7. Ritchie ME, Phipson B, Wu D, Hu Y, Law CW, Shi W, et al. limma powers differential expression analyses for RNA-sequencing and microarray studies. Nucleic Acids Res. 2015;43(7):e47.doi:10.1093/nar/gkv007

Supplementary Figures

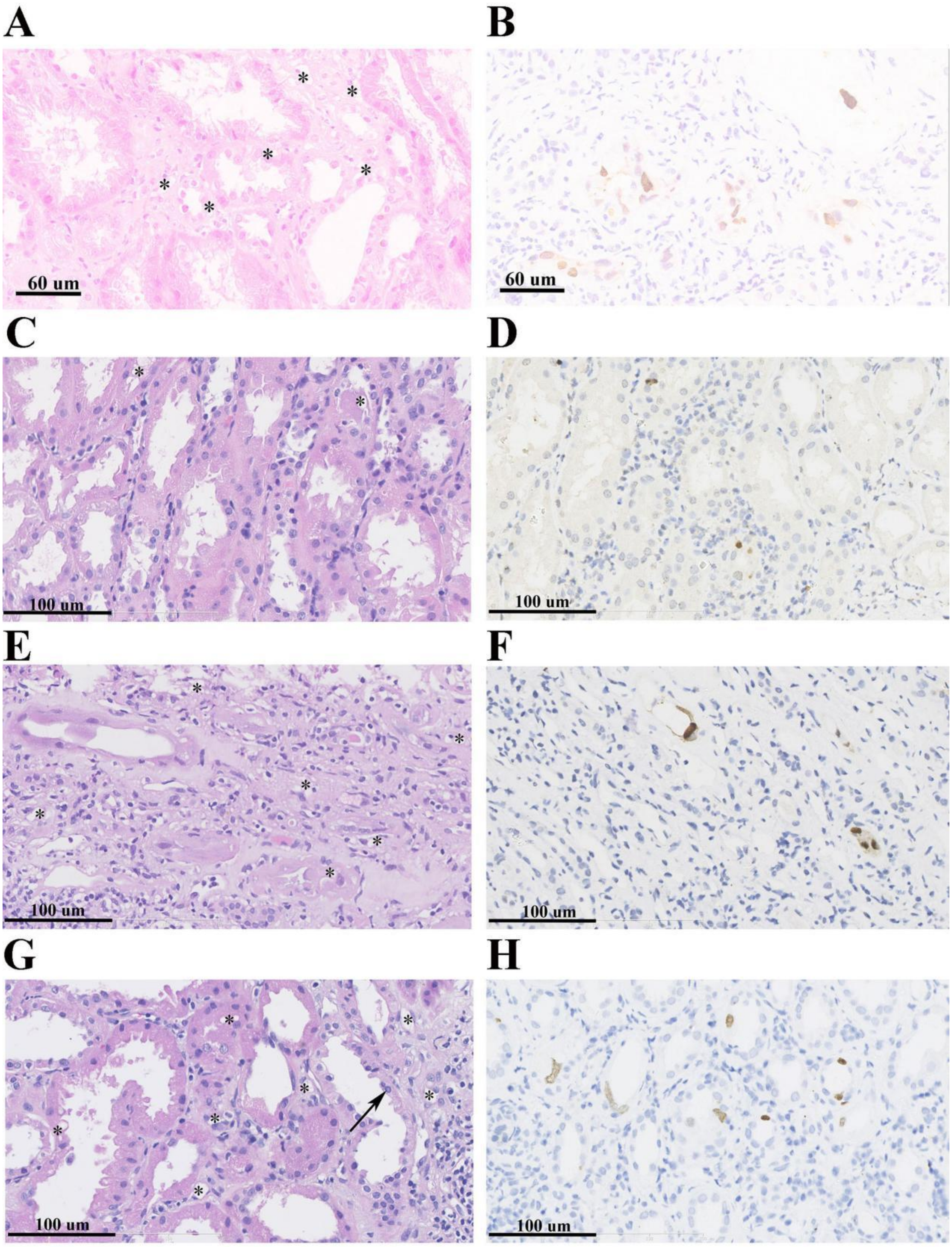

Figure S1. HE staining (A, C, E and G) and Immunohistochemistry staining (B, D, F and H) of BKPyVAN recipient 2 (A, B), 3 (C, D), 4 (E, F), 5 (G, H). The epithelial cells were swollen with large vacuole (*) within the cytoplasm and typical intranuclear inclusion (arrows) can be observed. In addition, anti-SV40 T-Ag positivity in tubular epithelial cell nuclei.

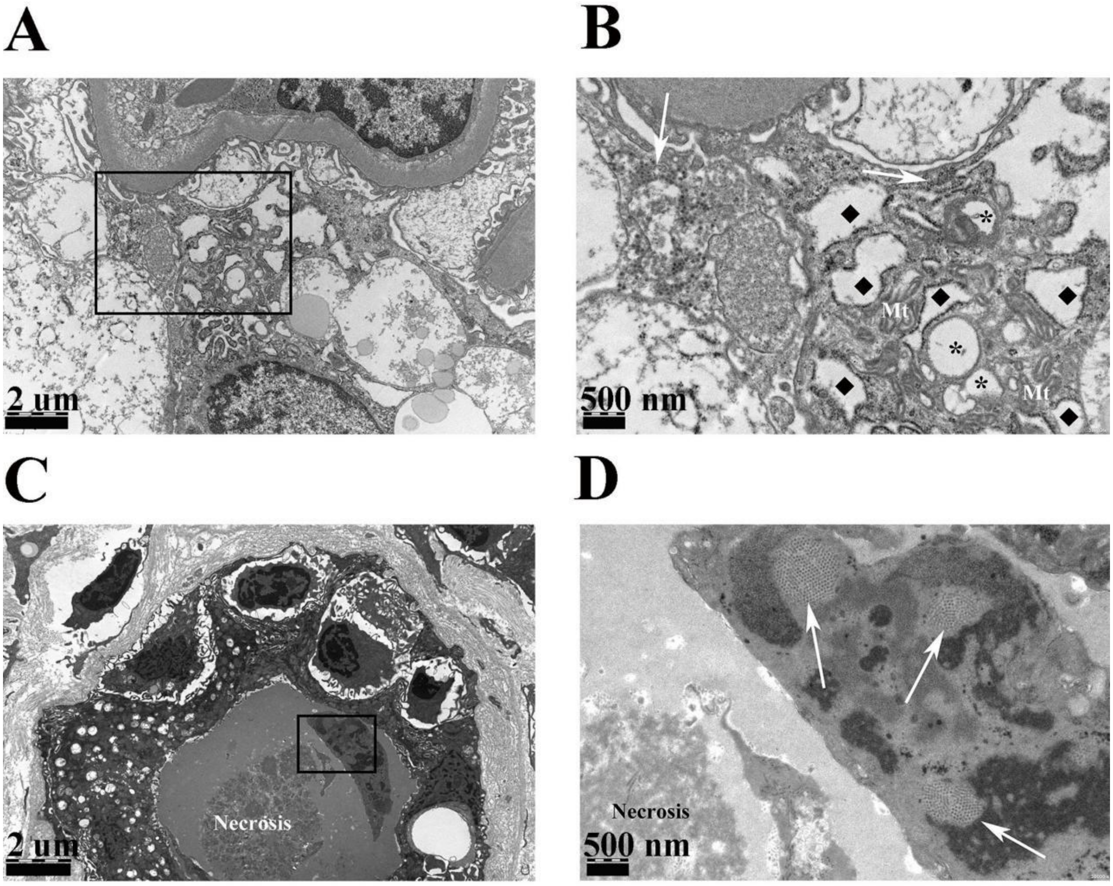

Figure S2. The electron micrographs of BKPyVAN recipient 3 （A, B） and BKPyVAN recipient 4 (C, D). The figures of the high-power field on the right (B, D) corresponded to the figures in the box of the low-power field on the left (A, C). The mild swelling of mitochondrion (Mt), virus particles (white arrows), cytoplasmic vacuolar degeneration (*) and expansion of rough endoplasmic reticulum (◆) can be observed in BKPyVAN. (A, C)×12000, (B,D)×30000.

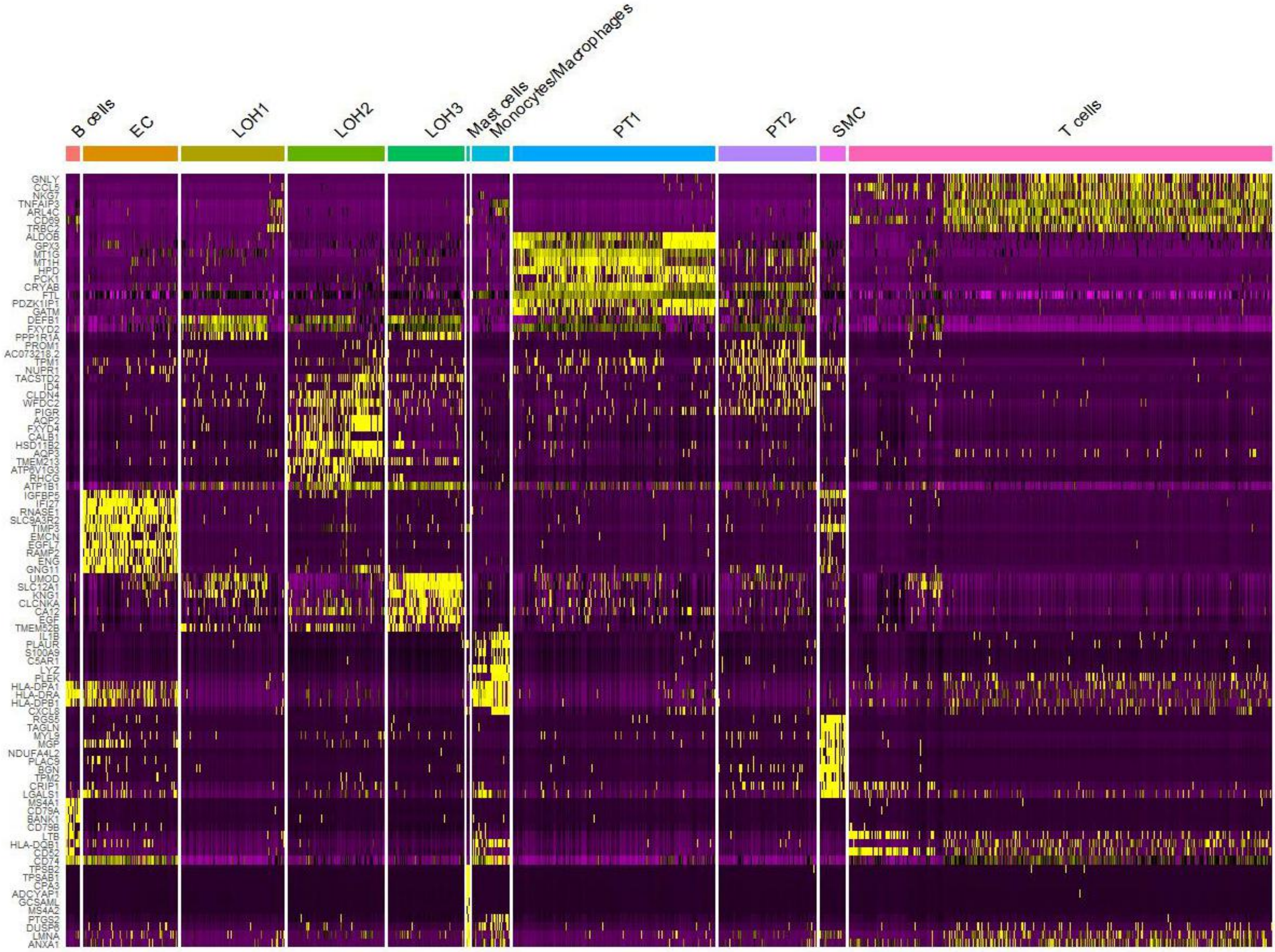

Figure S3. Heatmap showed the marker genes of all clusters and highlighted the selected marker genes for each cluster.

Supplementary Table 1

Table S1. Information on the samples

	Age / Sex	Smoker / Drinker	Height(cm) / Weight(kg)	Time after Surgery (months)	PRA		BKV DNA (copies/ml)		Creatinine at biopsy (umol/L)	Immnosuppression	Banff classification
					class I	class II	Urine	Blood			
BKPyVAN recipient 1	45 / male	N / N	173 / 72	16	(-)	1%	2.7*10^7	(-)	558	TAC / MMF / PSL	Stage B3
BKPyVAN recipient 2	36 / female	N / N	159 / 57	30	(-)	(-)	8.0*10^6	(-)	457	TAC / MMF / PSL	Stage B3
BKPyVAN recipient 3	38 / male	N / N	178 / 76	5	(-)	(-)	3.55*10^7	7.28*10^4	236	TAC / MMF / PSL	Stage B1
BKPyVAN recipient 4	31 / male	N / N	170 / 64	13	(-)	(-)	2.0*10^10	7.2*10^4	202	TAC / MMF / PSL	Stage C
BKPyVAN recipient 5	50 / female	N / N	158 / 41	21	2%	31%	8.61*10^8	9.65*10^3	242	TAC / MMF / PSL	Stage B3
stable transplanted recipients	48 / male	N / N	170 / 65	12	(-)	(-)	3.24*10^6	(-)	163	TAC / MMF / PSL	-
resolved BKVN	48 / male	N / N	160 / 41	28	(-)	(-)	2.79*10^6	(-)	174	TAC / MMF / PSL	-

PRA: panel reactive antibodies; TAC: tacrolimus; CyA: cyclosporine; MMF: mycophenolate mofetil; PSL: predonisolone.

Supplementary Table 2

Table S2. GSVA of PT

ID	score	index
ANTIGEN_PROCESSING_AND_PRESENTATION	41.87481877	186
NOD_LIKE_RECEPTOR_SIGNALING_PATHWAY	32.71270632	185
AMINOACYL_TRNA_BIOSYNTHESIS	30.61934581	184
TYPE_I_DIABETES_MELLITUS	30.31586297	183
LYSINE_DEGRADATION	28.10061372	182
TRYPTOPHAN_METABOLISM	25.84655625	181
CHEMOKINE_SIGNALING_PATHWAY	25.34295874	180
BUTANOATE_METABOLISM	24.93069802	179
AUTOIMMUNE_THYROID_DISEASE	24.39426513	178
BETA_ALANINE_METABOLISM	24.35173299	177
ALLOGRAFT_REJECTION	24.26903788	176
VIRAL_MYOCARDITIS	24.26007772	175
LIMONENE_AND_PINENE_DEGRADATION	24.069641	174
HISTIDINE_METABOLISM	23.83959165	173
GLYCEROLIPID_METABOLISM	23.77629546	172
GRAFT_VERSUS_HOST_DISEASE	23.34232759	171
PROPANOATE_METABOLISM	23.10880716	170
FATTY_ACID_METABOLISM	23.01198172	169
VALINE_LEUCINE_AND_ISOLEUCINE_DEGRADATION	22.91047687	168
GLYCOLYSIS_GLUCONEOGENESIS	22.9003887	167
ARGININE_AND_PROLINE_METABOLISM	22.86251324	166
RETINOL_METABOLISM	22.83470807	165
NEUROTROPHIN_SIGNALING_PATHWAY	20.88982957	164
ASCORBATE_AND_ALDARATE_METABOLISM	20.59453117	163
PYRUVATE_METABOLISM	19.79782995	162
NICOTINATE_AND_NICOTINAMIDE_METABOLISM	19.70188234	161
TOLL_LIKE_RECEPTOR_SIGNALING_PATHWAY	19.33703767	160
MAPK_SIGNALING_PATHWAY	19.23382853	159
DRUG_METABOLISM_CYTOCHROME_P450	18.78198119	158
ADIPOCYTOKINE_SIGNALING_PATHWAY	18.70671233	157
SYSTEMIC_LUPUS_ERYTHEMATOSUS	18.51734896	156
ALANINE_ASPARTATE_AND_GLUTAMATE_METABOLISM	18.12509033	155
PPAR_SIGNALING_PATHWAY	17.93823108	154
ENDOCYTOSIS	16.91491641	153
PRION_DISEASES	16.73468195	152
PROGESTERONE_MEDIATED_OOCYTE_MATURATION	16.45754368	151
GNRH_SIGNALING_PATHWAY	16.29845821	150
VASCULAR_SMOOTH_MUSCLE_CONTRACTION	16.23591933	149
STARCH_AND_SUCROSE_METABOLISM	16.23218445	148
TYROSINE_METABOLISM	16.0546985	147

INSULIN_SIGNALING_PATHWAY	15.8379058	146
PENTOSE_PHOSPHATE_PATHWAY	15.79002001	145
RENIN_ANGIOTENSIN_SYSTEM	15.75970094	144
ASTHMA	15.58835868	143
RIG_I_LIKE_RECEPTOR_SIGNALING_PATHWAY	15.02916372	142
NITROGEN_METABOLISM	15.01019554	141
T_CELL_RECEPTOR_SIGNALING_PATHWAY	14.91069759	140
CELL_ADHESION_MOLECULES_CAMS	14.80182154	139
DRUG_METABOLISM_OTHER_ENZYMES	14.76880419	138
PHOSPHATIDYLINOSITOL_SIGNALING_SYSTEM	13.77259452	137
RENAL_CELL_CARCINOMA	13.67527836	136
INOSITOL_PHOSPHATE_METABOLISM	13.67090998	135
CITRATE_CYCLE_TCA_CYCLE	13.55928122	134
GLYCINE_SERINE_AND_THREONINE_METABOLISM	13.28540862	133
METABOLISM_OF_XENOBIOTICS_BY_CYTOCHROME_P450	13.06959666	132
PATHWAYS_IN_CANCER	12.89134797	131
PROSTATE_CANCER	12.69835391	130
PATHOGENIC_ESCHERICHIA_COLI_INFECTION	12.59091802	129
EPITHELIAL_CELL_SIGNALING_IN_HELICOBACTER_PYLORI_INFECTION	12.47778359	128
FOLATE_BIOSYNTHESIS	12.47774812	127
LYSOSOME	12.38936528	126
REGULATION_OF_ACTIN_CYTOSKELETON	12.27575687	125
HEMATOPOIETIC_CELL_LINEAGE	12.08372185	124
JAK_STAT_SIGNALING_PATHWAY	12.06824661	123
OOCYTE_MEIOSIS	11.98315168	122
PEROXISOME	11.93571409	121
VALINE_LEUCINE_AND_ISOLEUCINE_BIOSYNTHESIS	11.8135218	120
LEUKOCYTE_TRANSENDOTHELIAL_MIGRATION	11.79699582	119
LONG_TERM_DEPRESSION	11.69325794	118
AMINO_SUGAR_AND_NUCLEOTIDE_SUGAR_METABOLISM	11.66483613	117
VASOPRESSIN_REGULATED_WATER_REABSORPTION	11.53172909	116
CYSTEINE_AND_METHIONINE_METABOLISM	11.3422399	115
OLFACTORY_TRANSDUCTION	11.3240302	114
GLIOMA	11.26892199	113
NATURAL_KILLER_CELL_MEDIATED_CYTOTOXICITY	11.20774944	112
CALCIUM_SIGNALING_PATHWAY	11.18699124	111
LEISHMANIA_INFECTION	11.16657031	110
PROXIMAL_TUBULE_BICARBONATE_RECLAMATION	10.76967032	109
TASTE_TRANSDUCTION	10.57862311	108
PANTOTHENATE_AND_COA_BIOSYNTHESIS	10.54288592	107
GAP_JUNCTION	10.39159544	106
APOPTOSIS	10.36790409	105
MELANOGENESIS	10.35580729	104

GLYOXYLATE_AND_DICARBOXYLATE_METABOLISM	10.29871342	103
PENTOSE_AND_GLUCURONATE_INTERCONVERSIONS	9.969462807	102
PHENYLALANINE_METABOLISM	9.87768548	101
B_CELL_RECEPTOR_SIGNALING_PATHWAY	9.762582569	100
ADHERENS_JUNCTION	9.695951539	99
VEGF_SIGNALING_PATHWAY	9.647304272	98
PRIMARY_BILE_ACID_BIOSYNTHESIS	9.645327618	97
AXON_GUIDANCE	9.608529598	96
TIGHT_JUNCTION	9.522362051	95
PROTEASOME	9.341878901	94
DORSO_VENTRAL_AXIS_FORMATION	9.334464405	93
FC_GAMMA_R_MEDIATED_PHAGOCYTOSIS	9.146451385	92
DILATED_CARDIOMYOPATHY	9.145560391	91
CELL_CYCLE	8.924840438	90
VIBRIO_CHOLERAE_INFECTION	8.817089696	89
SPHINGOLIPID_METABOLISM	8.600925546	88
BIOSYNTHESIS_OF_UNSATURATED_FATTY_ACIDS	8.401049313	87
SELENOAMINO_ACID_METABOLISM	8.334163573	86
WNT_SIGNALING_PATHWAY	8.28894296	85
CYTOSOLIC_DNA_SENSING_PATHWAY	7.927090679	84
CYTOKINE_CYTOKINE_RECEPTOR_INTERACTION	7.782875419	83
ACUTE_MYELOID_LEUKEMIA	7.687965128	82
LONG_TERM_POTENTIATION	7.683862028	81
ERBB_SIGNALING_PATHWAY	7.681424416	80
ARRHYTHMOGENIC_RIGHT_VENTRICULAR_CARDIOMYOPATHY_ARVC	7.539112693	79
PANCREATIC_CANCER	7.345602936	78
O_GLYCAN_BIOSYNTHESIS	7.343317534	77
FC_EPSILON_RI_SIGNALING_PATHWAY	7.092886256	76
GLUTATHIONE_METABOLISM	6.868285336	75
GLYCEROPHOSPHOLIPID_METABOLISM	6.761673894	74
STEROID_HORMONE_BIOSYNTHESIS	6.743318807	73
UBIQUITIN_MEDIATED_PROTEOLYSIS	6.509160532	72
GALACTOSE_METABOLISM	6.471753851	71
FOCAL_ADHESION	6.375933099	70
STEROID_BIOSYNTHESIS	6.352741742	69
CHRONIC_MYELOID_LEUKEMIA	6.32256691	68
ONE_CARBON_POOL_BY_FOLATE	6.317181771	67
COMPLEMENT_AND_COAGULATION_CASCADES	6.264270456	66
HYPERTROPHIC_CARDIOMYOPATHY_HCM	6.224399199	65
COLORECTAL_CANCER	6.176484014	64
TYPE_II_DIABETES_MELLITUS	5.923708216	63
HEDGEHOG_SIGNALING_PATHWAY	5.639623545	62
TERPENOID_BACKBONE_BIOSYNTHESIS	5.584957521	61

INTESTINAL_IMMUNE_NETWORK_FOR_IGA_PRODUCTION	5.547857785	60
SULFUR_METABOLISM	5.243766528	59
THYROID_CANCER	5.142271224	58
GLYCOSAMINOGLYCAN_BIOSYNTHESIS_KERATAN_SULFATE	5.108936126	57
RNA_DEGRADATION	4.70089269	56
NEUROACTIVE_LIGAND_RECEPTOR_INTERACTION	4.119752504	55
GLYCOSPHINGOLIPID_BIOSYNTHESIS_LACTO_AND_NEOLACTO_SERIES	3.532353944	54
NON_SMALL_CELL_LUNG_CANCER	3.421071236	53
N_GLYCAN_BIOSYNTHESIS	3.412625784	52
ARACHIDONIC_ACID_METABOLISM	3.164588756	51
MATURITY_ONSET_DIABETES_OF_THE_YOUNG	3.045473693	50
P53_SIGNALING_PATHWAY	2.963445915	49
SNARE_INTERACTIONS_IN_VESICULAR_TRANSPORT	2.8276351	48
SMALL_CELL_LUNG_CANCER	2.736028518	47
SPLICEOSOME	2.724365349	46
ETHER_LIPID_METABOLISM	2.533661561	45
MTOR_SIGNALING_PATHWAY	2.4553396	44
ALPHA_LINOLENIC_ACID_METABOLISM	2.451327812	43
TAURINE_AND_HYPOTAURINE_METABOLISM	2.138324055	42
GLYCOSPHINGOLIPID_BIOSYNTHESIS_GANGLIO_SERIES	1.93846668	41
NON_HOMOLOGOUS_END_JOINING	1.691172424	40
FRUCTOSE_AND_MANNOSE_METABOLISM	1.645342712	39
GLYCOSPHINGOLIPID_BIOSYNTHESIS_GLOBO_SERIES	1.283221833	38
BLADDER_CANCER	1.263475127	37
ENDOMETRIAL_CANCER	1.204674625	36
TGF_BETA_SIGNALING_PATHWAY	1.130077108	35
GLYCOSAMINOGLYCAN_DEGRADATION	0.644249575	34
MELANOMA	0.642186	33
REGULATION_OF_AUTOPHAGY	0.477024298	32
CIRCADIAN_RHYTHM_MAMMAL	0.386877079	31
OTHER_GLYCAN_DEGRADATION	0.272900909	30
AMYOTROPHIC_LATERAL_SCLEROSIS_ALS	0.264651119	29
PRIMARY_IMMUNODEFICIENCY	0.262451153	28
RIBOFLAVIN_METABOLISM	0.002052207	27
ECM_RECEPTOR_INTERACTION	-0.447724777	26
ABC_TRANSPORTERS	-0.657676703	25
LINOLEIC_ACID_METABOLISM	-0.869492262	24
HOMOLOGOUS_RECOMBINATION	-0.886077748	23
BASE_EXCISION_REPAIR	-1.157266255	22
NOTCH_SIGNALING_PATHWAY	-1.335169134	21
BASAL_TRANSCRIPTION_FACTORS	-1.48077165	20
BASAL_CELL_CARCINOMA	-1.650836196	19

MISMATCH_REPAIR	-1.804991059	18
ALZHEIMERS_DISEASE	-2.519332397	17
OXIDATIVE_PHOSPHORYLATION	-2.806355699	16
GLYCOSAMINOGLYCAN_BIOSYNTHESIS_HEPARAN_SULFATE	-2.938870624	15
GLYCOSYLPHOSPHATIDYLINOSITOL_GPI_ANCHOR_BIOSYNTHESIS	-3.007122296	14
DNA_REPLICATION	-3.789363036	13
PORPHYRIN_AND_CHLOROPHYLL_METABOLISM	-4.196276731	12
NUCLEOTIDE_EXCISION_REPAIR	-4.20537583	11
PARKINSONS_DISEASE	-5.049174006	10
PROTEIN_EXPORT	-5.176096537	9
PURINE_METABOLISM	-5.592148497	8
GLYCOSAMINOGLYCAN_BIOSYNTHESIS_CHONDROITIN_SULFATE	-5.637588447	7
HUNTINGTONS_DISEASE	-5.737814371	6
PYRIMIDINE_METABOLISM	-6.337690705	5
ALDOSTERONE_REGULATED_SODIUM_REABSORPTION	-8.545995926	4
RNA_POLYMERASE	-14.52948763	3
CARDIAC_MUSCLE_CONTRACTION	-26.37444812	2
RIBOSOME	-42.46509437	1
